# Fisheries impacts on China's coastal ecosystems: Unmasking a pervasive ‘fishing down’ effect

**DOI:** 10.1371/journal.pone.0173296

**Published:** 2017-03-07

**Authors:** Cui Liang, Daniel Pauly

**Affiliations:** 1 College of Ocean and Earth Sciences, Xiamen University, Xiamen, Fujian Province, China; 2 *Sea Around Us*, Institute for the Ocean and Fisheries, University of British Columbia, Vancouver, British Columbia, Canada; National Taiwan University, TAIWAN

## Abstract

Intensive fishing can strongly impact marine ecosystems; among other things, it usually causes the mean trophic level of the catches to decline, an indicator of the occurrence of the ‘fishing down’ (FD) phenomenon. Although FD occurs throughout the world oceans, it can easily be masked by diverse factors, which has misled authors as to its generality. In this contribution, which uses the East China Sea as an example, we explore the masking effect on FD of the taxonomic coarseness of catch data, of assuming that individual sizes remain constant after intensive fishing, and the geographic expansion of fisheries. The result showed that all of these masking factors occur in the East China Sea, where only a few species are reported separately and the bulk of the catch is pooled into non-informative ‘mixed fishes’. Also, the small mesh sizes and intensive fishing have reduced the sizes of fish and their trophic levels, while the fisheries have expanded offshore. Overall, taking the masking factors into account, the fishing down effect, i.e., the decline of the mean trophic level of the catch between 1979 and 2014 is in the order of 0.15 TL per decade, i.e., one of the highest estimates of FD in the world. Some ecological implications are presented.

## Introduction

Fishery activities impact not only the target and bycatch species, but also the whole marine ecosystems [1], such as destroying natural habitat, decreasing biomass, and reducing biodiversity [2]. In 2004, the Convention on Biological Diversity (CBD) identified the Marine Trophic Index as an indicator of the biodiversity of large fishes, and by implication, of the impact of fisheries on marine ecosystems [3]. The marine trophic index (MTI) is the CBD’s name for the mean trophic level (MTL) of fisheries catches, shown by Pauly et al. [4] to decline in many fisheries of the world, a phenomenon they called ‘fishing down marine food webs’ (FDMW). The fishing down effect means that the intensive fishing pressure leads to changes in catch compositions, which shifts from a dominance of large, high-trophic level species to relatively small, low-trophic level species. A large number of regional and national studies have confirmed the occurrence of fishing down effect, such as in Greece [5], Canada [6], Iceland [7], Uruguay [8], India [9], and others (see www.fishingdown.org). In spite of Branch et al. [10], who contested the ubiquity and even the very existence of FDMW, the phenomenon continues to be documented for various area of the world, e.g. Western Europe [11], and is now perceived as the widespread phenomenon that it is, as also reflected in the large number of citations that its original description, by Pauly et al. [4], continues to receive [12, 13].

However, there are issues regarding FDMW, and the main one is that it can be masked by extraneous factors [14], and mislead unwary critics, such as Caddy et al. [15], or Branch et al. [10]. Here, we investigate three of these masking effects, (1) taxonomic coarseness, or overaggregation, (2) size-related effects and (3) geographic expansion of the fisheries, using for all three effects catches and associated data from the People’s Republic of China, i.e., from the East China Sea.

The People’s Republic of China (hereinafter referred to as ‘China’) has the highest fisheries catches in the world [16], which plays an important role in its economic and societal development. The intensive fishery activities have caused profound changes in the marine ecosystem and preliminary analyses have shown a decline of the mean trophic level of its catches [17, 18]. However, the fishing down effect might still be underestimated because of the masking effects mentioned above. In this study, we use China as an example to illustrate the masking effect of these three factors on manifestations of the FDMW occurring along the East China Sea coast.

## Materials and methods

### Catch time series

**Fig 1:**
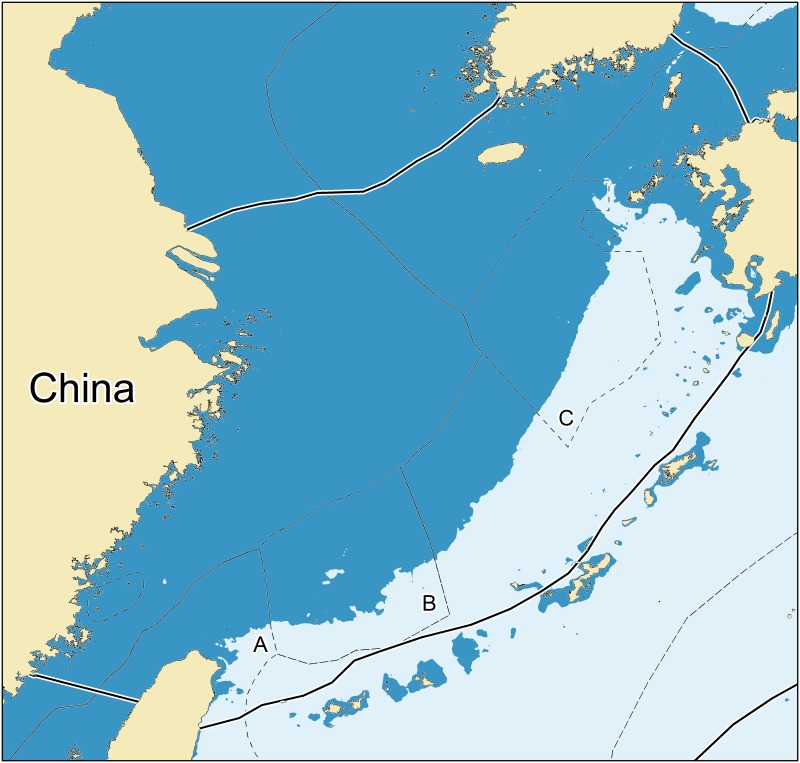
Map of East China Sea Large Marine Ecosystem (LME) as defined in Heileman and Tang [19], covering an area of over 744,000 km^2^, of which nearly 568,000 km^2^ is shelf, i.e., less than 200 m deep (from www.seaaroundus.org). The dotted lines are parts of Exclusive Economic Zones (EEZs) contested between various countries or areas (A: contested between China and Taiwan; B and C: contested between China and Japan).

The East China Sea is a marginal sea located in Western Pacific and is bordered by the central part of the Chinese mainland, the southern part of the Japanese Archipelago, and the northern coast of Taiwan (Fig 1). The East China Sea has abundant fishery resources, notably in its Zhoushan and Lüsi fishing grounds. The fish and other living resources in the East China Sea have been heavily exploited, with about 200 species being commercially fished [19].

The landings data used here are the fisheries catches in the East China Sea from 1979 to 2014, which originate from *China Fishery Statistical Yearbook* (CFSY) [20], in which fisheries statistics are presented as annual marine catches of major commercial fish species by the fleets of different provinces or province-level municipalities in China’s seas. These catch data pertain to various fishing gears (i.e., trawl, purse seine, gill net, stow net, etc.) at different scales (ranging from small scale fisheries to industrial fishery activities), but which were analyzed jointly.

In this contribution, we used the catches of three East China Sea coastal provinces or province-level municipality, i.e., Zhejiang, Fujian and Shanghai, to represent Chinese fisheries catches in the East China Sea. This data set consists of 22 species, as well as 5 genera of fishes (i.e., the small catches of invertebrates were omitted) and is presented in full in S1 Table. There are missing data in the catch for certain species in the early years in the data set, which is an inevitable problem for long time series statistical data. However, since *China Fishery Statistical Yearbook* only records major commercial species, the missing data suggests a relatively unimportant role for species omitted in the early years, so the analysis should not be much affected by their absence.

### Trophic levels and MTI

The trophic level (TL) estimates of consumers used here were based on diet composition data in FishBase (www.fishbase.org) and the equation:

$$\text{T}{\text{L}}_{\text{i}}=1+\sum_{\text{j}=1}^{\text{n}}\left(\text{D}{\text{C}}_{\text{i}\text{j}}\cdot \text{T}{\text{L}}_{\text{j}}\right)$$ (1)

where TL_i_ represents the trophic level of species i; DC_ij_ represents the fraction of prey j in the diet of predator i; n represents the number of prey species in the diet of predator i; TL_j_ represents the trophic level of prey j. Note that for primary producers (phytoplankton) and detritus, the TL value is 1, by definition, and hence exclusive herbivores have a TL = 2, etc. For fish and other aquatic predators, the TL can take values between 2.0 and 5.0. Note that most consumers are omnivores, and have non-integer TL-values, and that here, the TLs values for genera or higher groups were obtained from the mean TL value of their component species.

The MTIs of fisheries catches were calculated for each year using:

$${\text{MTI}}_{\text{k}}=\;\sum_{\text{i}=1}^{\text{m}}\left({\text{TL}}_{\text{i}}\cdot {\text{Y}}_{\text{ik}}\right)/\sum_{\text{i}=1}^{\text{m}}{\text{Y}}_{\text{ik}}$$ (2)

where MTI_k_ is the estimated mean trophic level in year k; m represents the number of species in catch in year k; TL_i_ is the trophic level of the species i and Y_ik_ is the catch of species i in year k.

### Taxonomic coarseness or overaggregation

In nature, trophic levels vary seasonally, regionally between size groups and even individuals of the same species [21, 22]. However, we can safely assume that, other things being equal, fish of otherwise similar species will tend to have different diets [23, 24]. Thus, when the available catch time series data are aggregated into higher categories (genera, families, or guilds and ‘functional groups’), information, and particularly the FDMW signal will be lost [17].

Here, we simulated the effect of declining data quality by computing the slopes of declining MTI time series for the East China Sea for different level of taxonomic resolution, i.e., species (with the original data), genus, family and order. When grouping the data into higher taxa, we simply sum the catches of lower taxa to obtain the catch of the higher taxa, and the TLs values for genera or higher groups were obtained from the unweighted mean TL value of their component species. The point here is that while we have taxonomically coarse statistics; we can know catch amount precisely, but not the composition of this catch, and hence its precise trophic level.

### Changes of TLs and body size caused by fishing mortality

Caddy et al. [15] pointed out that the trophic level of a certain fish would be changing with size. To investigate this effect, Pauly et al. [6] used data from FishBase on the size and trophic levels of fishes (excluding herbivores), summarized as:

$$\text{TL}=3+\text{b}\cdot {\text{log}}_{10}(\text{L})$$ (3)

where 3 is the trophic level of a fish larvae feeding exclusively on (herbivorous) zooplankton, b_1_ = 0.24 for first-order carnivores (i.e., zooplanktivores, whose adult TL ranges from 2.75 to 3.75), and b_2_ = 0.63 for higher-order carnivores (with adult TL higher than 3.75) [6], and L represents the body size of fish species. The value of b would be negative for herbivores and detritivores, for which the adult TL = 2, but this case is not considered here, as we omitted the only organisms (i.e., invertebrates) that include herbivores (e.g., bivalves) or detritivores (e.g., crabs); see above.

Beverton and Holt [25] showed that if fishes grow according to the von Bertalanffy growth function (VBGF), then their instantaneous total mortality (Z) can be expressed as:

$$\text{Z}=\;\left[\text{K}\cdot \left({\text{L}}_{\infty }-\bar{\text{L}}\right)\right]/\left(\bar{\text{L}}{\text{-L}}_{\text{c}}\right)$$ (4)

where L_∞_ is the asymptotic length, i.e., the mean length the fish would reach if they were to grow forever; K is a curvature parameter (of dimension time^-1^); L_c_ is the size at first capture, and $\bar{\text{L}}$ is the mean length computed from L_c_ upward.

Considering that Z is the sum of natural mortality (M) and fishing mortality (F), and re-expressing exploitation rate (E = F/Z) as E = 1-(M/K)/(Z/K), Pauly and Soriano [26] rearranged Eq 4 to:

$$\bar{\text{L}}=\;\left[{\text{L}}_{\infty }\text{+}\left(\frac{\text{M/K}}{\text{1-E}}\;\cdot {\text{L}}_{\text{c}}\right)\right]/\left[\frac{\text{M/K}}{\text{1-E}}+1\right]$$ (5)

Combining Eqs 3 with 5, the change in TLs associated with an increase in fishing pressure (as expressed by increasing E) can then be estimated from:

$$\Delta \text{TL}=\text{b}\cdot {\text{log}}_{10}\left[\frac{\left({\text{L}}_{\infty }+\frac{\text{M/K}}{\text{1-E}}\;\cdot {\text{L}}_{\text{c}}\right)/(\frac{\text{M/K}}{\text{1-E}}+1)}{\left({\text{L}}_{\infty }+(\text{M/K}\right)\cdot {\text{L}}_{\text{c}})/\left(\left(\text{M/K}\right)+1\right)}\right]$$ (6)

With b = b_1_ for first-order carnivores and b_2_ = b for higher-order carnivores.

Authors of this contribution estimated growth parameters (L_∞_, K), L_c_, M and F in 2000s for 10 commercially exploited fishes in China’s seas [27] (see S2 Table), including five species considered here, i.e., *Larimichthys polyactis*, *Trichiurus lepturus*, *Scomber japonicus*, *Decapterus maruadsi* and *Engraulis japonicus*. We used the estimated parameters for these five species, while for the other species or genera, we extracted growth parameters (L_∞_, K) from FishBase, and estimated M from the empirical formula of [28]:

$$\text{log}\left(\text{M}\right)=\;-0.0066-0.279\cdot \text{log}\left({\text{L}}_{\infty }\right)+0.6543\cdot \text{log}\left(\text{K}\right)+0.4634\cdot \text{log(T)}$$ (7)

where L_∞_ is expressed in cm, K in year^-1^,and T is the mean annual water temperature to which the fish are exposed, in °C. We used here the temperature reported by Belkin [29] for the East China Sea. Given the unavailability of more recent data, we also used the mean temperature from 2000 to 2006 for the years 2007 to 2014.

The mean of F in 2000s (i.e., F = 1) for ten species in S2 Table was used for the estimation of the fishing mortality to which the other East China Sea taxa are exposed. Since F has a linear relationship to catches (Y), i.e. Y = F·B, with B as the biomass, which is assumed constant. This assumption is justified based on the fact that the initial depletion of accumulated biomass, in the East China Sea, occurred before 1979, and that reported catch/effort data for the East China Sea were remarkably stable throughout the 1980s and early 1990s [30]. Thus, we calculated F in every year according to the ratios of catches in that year to the mean catches in 2000s, and the exploitation rate (E) was computed from E = F/(M+F). Discounting the aberrantly thin cutlassfish (*Trichiurus lepturus*), the mean value of L_c_ of the species in S2 Table i.e., 5.8 cm, was taken as estimate of L_c_ of the other 21 species or genera, except for snake-like daggertooth pike conger (*Muraenesox cinereus*), for which L_c_ was set at 10 cm.

We calculated change in TLs according to Eq 6 for each species in every year associated with an increase in fishing pressure (E), and subtracted the resulting estimate of ΔTL from the annual estimates of the MTI to estimate the specific effect of fishing on TL via size reduction.

### Accounting for the spatial expansion of fisheries

#### The Fishing-in-Balance (FiB) index and its spatial extension

The MTI computations are most robust when the available catch series pertain to a well-defined area, where the full spectrum of species is accessible from the onset of a fishery. However, in most fisheries, when costal abundance decline, the fisheries that cause the decline expand further offshore, into deeper waters, thus catching previously under- or unexploited species, with the expansion extended when the new resource, again, become depleted [31, 32].

This process, which obviously will (at least partly) mask FDMW, can be detected, however, by the Fishing-in-Balance (FiB) index of Pauly et al. [33], defined as:

$${\text{FiB}}_{\text{k}}={\text{log}}_{10}\left[{\text{Y}}_{\text{k}}\cdot {\left(\frac{\text{1}}{\text{TE}}\right)}^{{\text{MTI}}_{\text{k}}}\right]-{\text{log}}_{10}\left[{\text{Y}}_{0}\cdot {\left(\frac{\text{1}}{\text{TE}}\right)}^{{\text{MTI}}_{0}}\right]$$ (8)

where Y_k_ and MTI_k_ are the reported catches and marine trophic index in year k; Y_0_ and MTI_0_ represent the reported catches and marine trophic index in the initial year, and TE is the transfer efficiency between trophic levels, estimated as 0.1 by Pauly and Christensen [34].

The FiB index is defined such that, assuming, e.g., TE = 0.1, a decrease of 1 TL should correspond to a 10-fold catch increase, while a TL increase of 1 should corresponds to a 10-fold decrease in catch; finally, the FiB should maintain its value (remains = 0) when a decline in MTI goes along with an ecologically corresponding catch increase, or vice-versa. When however, catches increase more than expected from the MTI decrease (and the estimate of TE is thought to be appropriate for the ecosystem in question), this will result in predicted FiB increases according to Eq 7, and imply that the surplus catch originates from a nearby ecosystem, into which the fishery has expanded.

This offshore movement can be detected when the new catches exceed the catch what would be expected given overall mean trophic level (i.e., the MTI) of the catches and the transfer efficiency (TE) between trophic levels.

This implied expansion of the fishery can be made explicit, in term of the area covered by the fishery by defining an expansion factor (EF) for every k, i.e.

$${\text{EF}}_{\text{k}}=10^{{\text{FiB}}_{\text{k}}}$$ (9)

whose assumptions and caveats are presented in Bhathal and Pauly [9].

#### The Region-based Marine Trophic Index (RMTI)

The indicators presented can diagnose the masking effects of fisheries expansion on FDMW, but cannot correct for it. A correction can be achieved, however, by combining the logic of Eq 2 with that of Eq 8 to define another indicator, the region-based MTI (RMTI) of Kleisner et al. [35].

When calculate RMTI, we assume that fisheries is expanded in a sequential manner that one fishing region is saturated before moving onto the next. This assumption is reasonable, because considering the high cost of offshore fisheries, fishing activities tend to be confined inshore before the inshore fishing region become overexploited, and the fishing fleets head to the next, offshore fishing grounds. When fishing in a given area is conducted such that FiB = 0, and given an initial catch Y_0_ and initial mean trophic level MTI_0_, then the reported catch Y_k_ can be deduced by Eq 8:

$${\text{Y}}_{\text{k}}={\text{Y}}_{0}\cdot {\left(\frac{\text{1}}{\text{TE}}\right)}^{{\text{MTI}}_{0}{\text{-MTI}}_{\text{k}}}$$ (10)

Therefore, any reported catch ${\text{Y}}_{\text{k}}>{\text{Y}}_{0}\cdot {\left(\frac{\text{1}}{\text{TE}}\right)}^{{\text{MTI}}_{0}{\text{-MTI}}_{\text{k}}}$ indicates that geographic expansion occurred.

Eq 10 relies entirely on the initial mean trophic index (MTI_0_), which, however, may have not been based from the onset on all species in the ecosystem. To correct for this, a series of possible trophic levels instead of one MTI_0_ may be used, with MTI_0_ acquiring any value in the range of [TL_lower_, TL_upper_], where TL_lower_ and TL_upper_ are the lowest and highest TL in the catches, respectively. By assigning the range [TL_lower_, TL_upper_] into J trophic levels, for each trophic level j, a corresponding potential catch pY_kj_ can be calculated as:

$${\text{pY}}_{\text{kj}}={\text{Y}}_{0}\cdot {\left(\frac{\text{1}}{\text{TE}}\right)}^{{\text{TL}}_{\text{j}}{\text{-MTI}}_{\text{k}}}$$ (11)

and the total maximum potential catch in year k can be obtained from:

$${\text{pY}}_{\text{k}}=\sum_{\text{j}=1}^{\text{J}}\left({\text{pY}}_{\text{kj}}\cdot \text{Pr}\left({\text{TL}}_{\text{j}}\right)\right)$$ (12)

where Pr (TL_j_) is the probability that MTI_0_ = TL_j_. as detailed information on the distribution of trophic levels in East China Sea is unavailable, we used a uniform probability distribution; thus, pY_k_ is here the average of pY_kj_. The potential catch pY_k_ is independent of the initial mean trophic level (MTI_0_) and represents the maximum catch a fishery could extract from a distinct region, given the value of TE. Any reported catch Y_k_ that exceed pY_k_ indicates geographic expansion, and the corresponding year k indexed by node (n_r_), where r represents each identified new region.

In any year k that follows n_r_ (k>n_r_), the catches and associated MTI can then be calculated separately for different regions, under the assumptions and caveats presented in Kleisner et al. [35].

**Fig 2:**
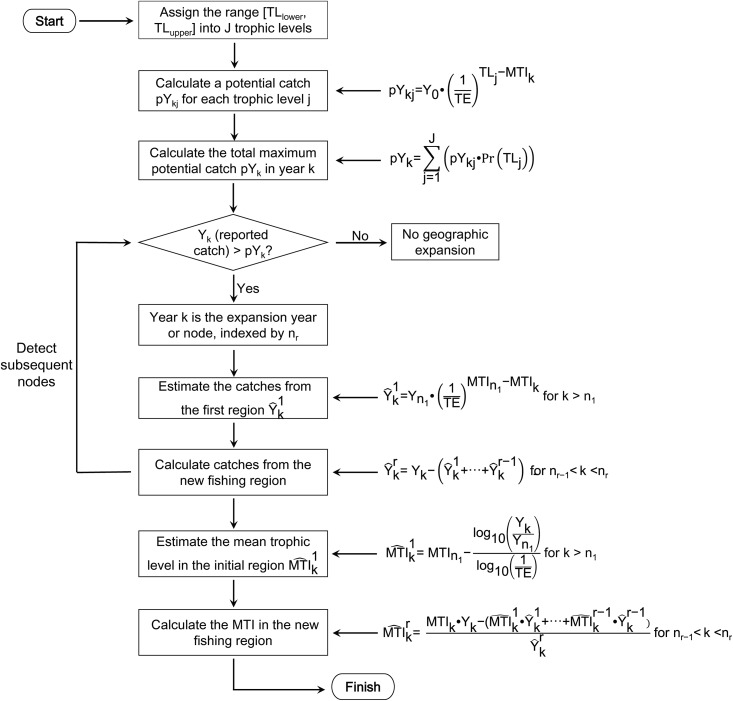
Conceptual representation and key equations of the approach to compute RMTI time series. This can be implemented through a program in R (available at http://www.seaaroundus.org/regional-mti-tools/) or an Excel spreadsheet available from the first author.

A conceptual representation of the RMTI computation and the equations in each step are illustrated in Fig 2. In principle, given a sufficiently long time catch time series, several regions (which can be conceived as parallel coastal strips, each with a separate FDMW trend) could be identified by this method, but 3 regions appear to be the maximum [35].

## Results

### Main features of the catch time series

**Fig 3:**
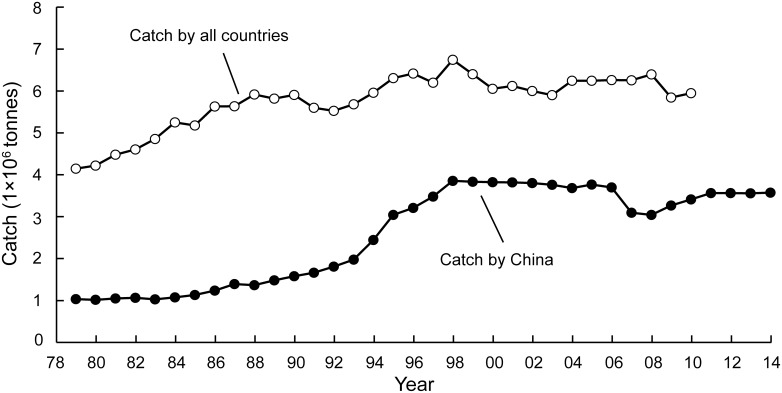
Time series of catches from the East China Sea Large Marine Ecosystem, by China (from successive *China Fishery Statistical Yearbook*) and by all countries whose fleets operate in that ecosystem (from www.seaaroundus.org).

Fig 3 presents the Chinese catch reported from the East China Sea Large Marine Ecosystem (see S1 Table for the corresponding tabular data), jointly with the larger catch also extracted by Japan, South Kora and Taiwan (see Pauly and Zeller [36]; Zeller et al. [37], and www.seaaroundus.org). As might be seen from Fig 3, from 1979 to the beginning of 1990s, the Chinese catches were relatively low. However, they strongly increased starting in the early to mid-1990s, then stabilized from the late 1990s on.

### The MTI and the effects of taxonomic overaggregation

**Fig 4:**
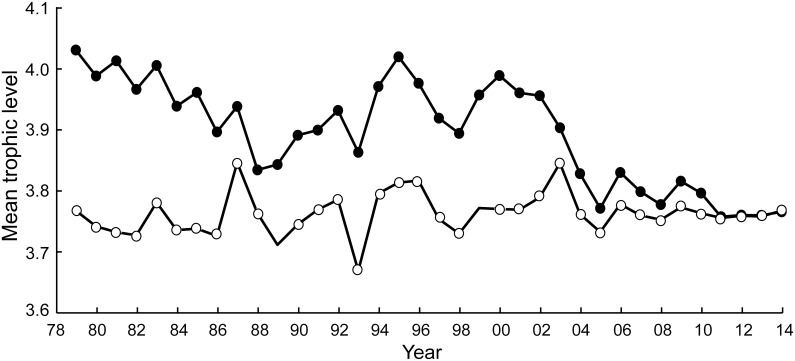
Time series of the mean trophic levels of Chinese catch data from the East China Sea. Black dots: original data, consisting of 22 species, as well as 5 genera. The slope of a regression line (not shown) fitted to this time series is -0.0067, corresponding to a significant (p<0.05) decline of 0.074 TL per decade. Open circles: the same catch, aggregated by Order (or higher groupings), and showing no declining trend.

The different taxa in Chinese catch data for the East China Sea, when associated with the trophic level estimates in S3 Table, suggested a strong fishing down effect in East China Sea, with the mean trophic level decreasing from over 4.0 to below 3.8in the years from 1979 to 2014 (Fig 4). This corresponded to a fishing down slope of -0.0067. However, when these catch data were aggregated into higher taxa, the decline became less pronounced, with the downward trend disappearing completely when the catch data were grouped into orders (Fig 4).

### The effects of size

**Fig 5:**
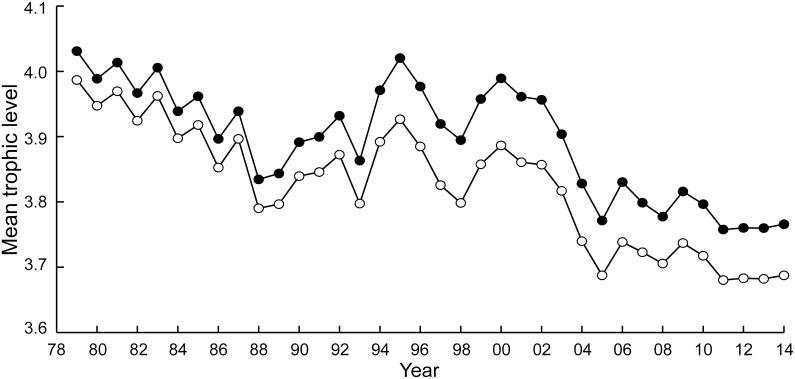
Fishing down trends in the Chinese fisheries of the East China Sea. Black dots: ignoring body size effects. The slope of a regression line (not shown) fitted to this time series is -0.0067, corresponding to a significant (p<0.05) decline of 0.074 TL per decade (as in Fig 4). Open dots: taking the effect of fishing on fish size, and hence on trophic level, into account. The slope of a regression line (not shown) fitted to this time series is -0.0076, corresponding to a significant (p<0.05) decline of 0.083 TL per decade (see text).

With body size positively correlated with trophic level, accounting for the response of body size to increased fishing pressure intensifies the decline of trophic level from 1979 to 2014 (Fig 5). In 1979, with a relatively lower exploitation rate, the MTI estimates taking body size change into account was 0.04 lower than estimated by not taking body size changes into account. In the early 2010s, that difference was 0.8–0.10. The corresponding slopes are -0.0067 without, and -0.0076 with size-related effects, i.e., 0.074 vs 0.083 TL per decade.

### Region-based marine trophic index

**Fig 6:**
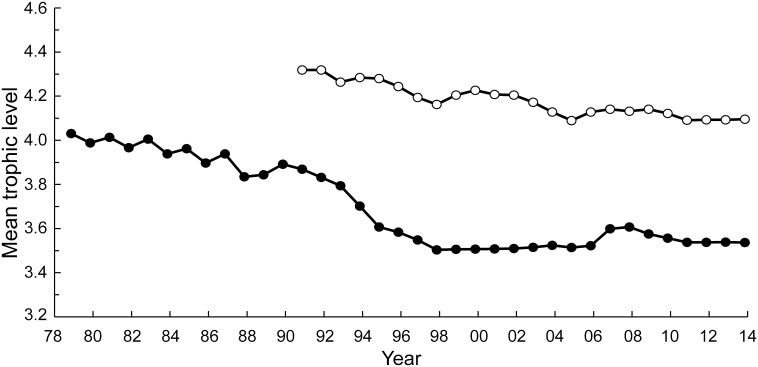
Fishing down trends in the Chinese fisheries of the East China Sea, taking into account the offshore expansion of the fisheries using the method of Klesner et al. [35]. Black dots: MTI trend in nearshore region; the slope of a regression line (not shown) fitted to this time series is -0.0165, corresponding to a significant (p<0.05) decline of 0.137 TL per decade. Open circle: MTI trend in the offshore region; the slope of a regression line (not shown) fitted to this time series is -0.0098, corresponding to a significant (p<0.05) decline of 0.093 TL per decade.

Applying the method of Kleisner et al. [35] for computing trophic levels unaffected by geographic expansion to the catch data of S1 Table led, with the TL by taxa in S3 Table, to Fig 6. This figure suggests that from 1991, Chinese vessels began to exploit an (offshore) region other than the more coastal region where they had operated before (and which continued to be exploited). Along the coast, the RMTI declined from over 4.0 in 1979 to less than 3.6 in 2014, corresponding to a TL decrease of 0.137 per decade. In contrast, in 1991, the mean trophic level of offshore catches was over 4.3 and 4.1 in 2014, corresponding to a decline of about 0.1 TL per decade (Fig 6).

## Discussion

**Fig 7:**
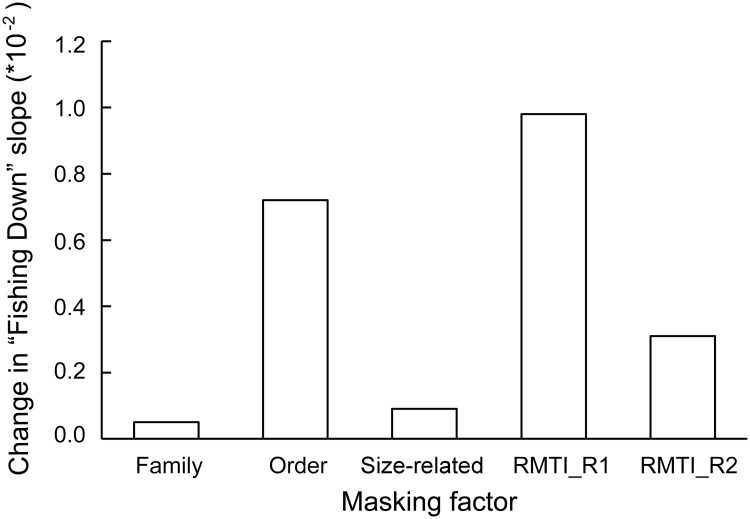
Comparing the effect of three masking factors on the estimated strength of ‘fishing down’ in the East China Sea, 1979 to 2014. The histograms for ‘Family’ and ‘Order’ quantify the decline that is masked by grouping all the catch data at the corresponding taxonomic level (There were only two species in the catch data used here which belonged to a same genus; hence the effect of genus as an aggregation level could not be detected here; it is likely to be smaller than that of family). ‘Size-related’ refers to the trophic level reduction due to the decline in body sizes, i.e., mean length, with fishing intensity. The last two histograms, ‘RMTI_R1’ and ‘RMTI_R2’ refer to the masking effect of offshore expansion of the fishery. Clearly, relying on coarse data, aggregated at the level of Orders or above, and not taking account of the offshore expansion of fisheries will mask of fishing down, at least in part, as occurring in the East China Sea (see text).

While Pauly et al. [38] were the first to examine masking effects to fishing down, in response to Caddy et al. [15], and subsequent contributions examined the potential effect of various masking factors in various areas [6, 7, 17]; the present contribution is the first to compare the strength of making factors in a given area, here the East China Sea (Fig 7), and to sum them, to about 0.15 TL per decade (depending on which factor is counted).

Caddy et al. [15] claimed that the decrease in MTI reported by [4] might be an artifact due to their use of overagreegated catch data, i.e., the global catch data compiled and disseminated by FAO [39]. On the contrary, Pauly et al. [38] suggested, and Pauly and Palomares [17] confirmed that aggregating catch time series into higher taxonomic grouping would gradually reduce, then completely mask the down sloping fishing down signal. This is here confirmed for the East China Sea (Fig 4), which suggest that the ‘starting’ estimate of fishing down in that LME, of 0.074 per decade is underestimated. Indeed, reporting overaggregated taxa is a persistent issue with China’s statistical reporting system, at least as compared to countries such as the Republic of Korea, Japan, the United States of America, Canada, Iceland and Norway (Table 1).

**Table 1:** Taxonomic precision of national catch data reported by various countries to the FAO (Based on FAO data for 1950–2014).

Taxon	China	Korea	Japan	USA	Canada	Iceland	Norway
**Species**	57.1	63.2	67.4	75.3	71.2	76.4	76.0
**Genus**	4.8	3.9	5.7	4.6	5.0	5.5	4.6
**Family**	10.5	9.7	6.4	7.0	5.8	3.6	5.7
**Subtotal**	72.4	76.7	79.4	86.9	82.0	85.5	86.3
**Order**	1.0	3.9	2.5	2.1	2.9	2.7	1.7
**Class**	0.0	1.9	1.1	0.6	0.7	0.9	1.1
**Phylum**	1.0	0.8	1.0	0.6	0.0	0.0	0.6
**‘Mix’**	25.6	16.7	16.0	9.8	14.4	10.9	10.3

However, the effect of overaggregation remains small when similar fishes (i.e., congenerics, or even belongings to the same family) are pooled, presumably because they tend to be of similar sizes, and hence consume prey of similar sizes and trophic levels.

Size itself, which Caddy et al. [15] also thought would generate artifact appears to have a small effect on the strength of the fishing down effect, at least when compared to the high levels of overaggregation one is likely to encounter in practice (Fig 7). Thus, in spite of the intense fishing off the Chinese coast, leading to a ‘miniaturization’ of resource species [40], the impact on the rate of TL decline is modest. This result, which confirms earlier findings by Pauly et al. [6] and Valtýsson and Pauly [7] also suggest that the detailed information required to parametrize Eq 6 (i.e., to quantify size effects) may not be worth assembling, at least in cases for routine examinations of the status of fisheries.

On the other hand, Fig 7 clearly shows that taking account of the geographic expansion of fisheries is absolutely necessary when studying fishing down effects. Not taking account of this expansion caused Branch et al. [10] to misinterpret apparent TL increase as recovery of the higher-trophic level species targeted expanding fisheries [35]. While such gross error will remain rare, the fact remains that any estimate of the strength of the fishing down effect will be underestimated when fisheries expand—which most of them do [41].

In China, the intensification of bottom trawling and innumerable stake nets had depleted the inshore resources, and induced the collapse of several species [42]. In response, the Chinese authorities called for a further “acceleration of fishery development through structure adjustment” [43], i.e., for a shift of fishing effort from inshore to offshore, and also offered technical support and subsidies to offshore fisheries [44], which also resulted in Chinese distant-water fleets that began operating in the mid-1980s [43, 45]. To protect the coastal resources, from the mid-1980s on, the Chinese government announced a series of policies to control inshore fishing effort, including summer closures of 2–3 months.

However, these measures have not reduced the intense fishing pressure in coastal areas, as manifested in the disappearance of numerous species from catches, the “miniaturization” of those remaining, and the fact that an increasing fraction of the catch is unfit for human consumption [46]. Such landings find a ready market in China, which has an enormous aquaculture industry in need of feeds. However, this causes animal protein losses which would be avoided if more of the catch landed by Chinese domestic fisheries could be directly consumed by people.

We point out finally, that the MTI is an index of the biodiversity of large, high-tropic level predators, and that a low MTI, in a given marine ecosystem, suggest that top-down control has been replaced by bottom-up control [47], with all that this implies in term of harmful algal blooms, jellyfish outbreak, and other pathologies of disturbed ecosystems. Clearly, the aspirations of Chinese people ought to include reversing these trends.

## Supporting information

S1 Table Time series of Chinese catches (in tonnes) from the East China Sea Large Marine Ecosystem (from successive *China Fishery Statistical Yearbook*). (DOCX)

S2 Table Estimated growth and mortality parameters in 2000s for 10 commercially exploited fishes in China’s seas. (DOCX)

S3 Table Trophic levels for the studied 22 species and 5 genera. (DOCX)
