# Supplementary material for: Fisheries impacts on China's coastal ecosystems: Unmasking a pervasive ‘fishing down’ effect
Source: PLoS One. 2017 Mar 7;12(3):e0173296. doi: 10.1371/journal.pone.0173296 (PMC5340396; doi:10.1371/journal.pone.0173296)
Supplement: S1 Table — (DOCX) [file pone.0173296.s001.docx]

| **Common name** | **Scientific name** | **Year** | | | | | | | | |
| --- | --- | --- | --- | --- | --- | --- | --- | --- | --- | --- |
|  |  | **1979** | **1980** | **1981** | **1982** | **1983** | **1984** | **1985** | **1986** | **1987** |
| **Large yellow croaker** | *Larimichthys crocea* | 77897 | 77173 | 72502 | 48658 | 25163 | 35177 | 12134 | 9183 | 6655 |
| **Yellow croaker** | *Larimichthys polyactis* | 23128 | 13071 | 10557 | 7136 | 8206 | 6308 | 4750 | 6397 | 3337 |
| **Largehead hairtail** | *Trichiurus lepturus* | 355520 | 353532 | 390536 | 359092 | 339311 | 310737 | 325889 | 307210 | 285532 |
| **Pomfret** | *Pampus* | 22033 | 20857 | 19826 | 23018 | 18696 | 23993 | 31924 | 31872 | 44371 |
| **Elongate ilisha** | *Ilisha elongata* | 9323 | 8264 | 7816 | 6416 | 8210 | 8936 | 9857 | 6994 | 5107 |
| **Japanese Spanish mackerel** | *Scomberomorus niphonius* | 4750 | 5253 | 5277 | 8046 | 11582 | 16723 | 17762 | 18275 | 17612 |
| **Red seabream** | *Pagrus major* | 141 | 425 | 3877 | 690 | 906 | 857 | 637 | 1232 | 2300 |
| **Grouper** | *Epinephelus* | 338 | 391 | 556 | 695 | 1111 | 1602 | 1721 | 1761 | 2623 |
| **Daggertooth pike conger** | *Muraenesox cinereus* | 6540 | 8164 | 8272 | 10586 | 13339 | 15823 | 17843 | 24355 | 28051 |
| **Filefish** | *Thamnaconus* | 47582 | 99467 | 121547 | 136133 | 37394 | 120249 | 117232 | 162964 | 203856 |
| **Chub mackerel** | *Scomber japonicus* | 61771 | 47019 | 40384 | 68393 | 101613 | 79283 | 61254 | 90592 | 108544 |
| **Japanese scad** | *Decapterus maruadsi* | 34230 | 51371 | 34637 | 45276 | 69186 | 64604 | 80161 | 83199 | 152780 |
| **Japanese anchovy** | *Engraulis japonicus* | -- | -- | -- | -- | -- | -- | -- | -- | -- |
| **South American pilchard** | *Sardinops sagax* | -- | -- | -- | -- | -- | -- | -- | -- | -- |
| **Japanese barracuda** | *Sphyraena japonica* | -- | -- | -- | -- | -- | -- | -- | -- | -- |
| **Pacific herring** | *Clupea pallasii* | -- | -- | -- | -- | -- | -- | -- | -- | -- |
| **Golden threadfin bream** | *Nemipterus virgatus* | -- | -- | -- | -- | -- | -- | -- | -- | -- |
| **Pacific cod** | *Gadus macrocephalus* | -- | -- | -- | -- | -- | -- | -- | -- | -- |
| **Silver croaker** | *Pennahia argentata* | -- | -- | -- | -- | -- | -- | -- | -- | -- |
| **Yellow drum** | *Nibea albiflora* | -- | -- | -- | -- | -- | -- | -- | -- | -- |
| **Mi-iuy croaker** | *Miichthys miiuy* | -- | -- | -- | -- | -- | -- | -- | -- | -- |
| **Baby croaker** | *Collichthys* | -- | -- | -- | -- | -- | -- | -- | -- | -- |
| **Horsehead tilefish** | *Branchiostegus japonicus* | -- | -- | -- | -- | -- | -- | -- | -- | -- |
| **Pacific sandlance** | *Ammodytes personatus* | -- | -- | -- | -- | -- | -- | -- | -- | -- |
| **Tuna** | *Thunnus* | -- | -- | -- | -- | -- | -- | -- | -- | -- |
| **Japanese jack mackerel** | *Trachurus japonicas* | -- | -- | -- | -- | -- | -- | -- | -- | -- |
| **Flathead grey mullet** | *Mugil cephalus* | -- | -- | -- | -- | -- | -- | -- | -- | -- |
| **Subtotal** | | 643253 | 684987 | 715787 | 714139 | 634717 | 684292 | 681164 | 744034 | 860768 |
| **Mixed fish** | | 392585 | 331318 | 332541 | 348923 | 392128 | 390085 | 451755 | 491266 | 560516 |
| **Total catch** | | 1035838 | 1016305 | 1048328 | 1063062 | 1026845 | 1074377 | 1132919 | 1235300 | 1393233 |

**S1 Table. Time series of Chinese catches (in tonnes) from the East China Sea Large Marine Ecosystem (from successive *China Fishery Statistical Yearbook*)**

| **Common name** | **Scientific name** | **Year** | | | | | | | | |
| --- | --- | --- | --- | --- | --- | --- | --- | --- | --- | --- |
|  |  | **1988** | **1989** | **1990** | **1991** | **1992** | **1993** | **1994** | **1995** | **1996** |
| **Large yellow croaker** | *Larimichthys crocea* | 4165 | 1462 | 1456 | 1871 | 2179 | 1686 | 8665 | 23195 | 16105 |
| **Yellow croaker** | *Larimichthys polyactis* | 2774 | 2561 | 3840 | 7950 | 14336 | 13481 | 23674 | 34472 | 48789 |
| **Largehead hairtail** | *Trichiurus lepturus* | 254284 | 284203 | 329599 | 369740 | 419128 | 417096 | 540149 | 703982 | 612960 |
| **Pomfret** | *Pampus* | 33564 | 35792 | 37247 | 34777 | 31828 | 41936 | 52567 | 103820 | 99871 |
| **Elongate ilisha** | *Ilisha elongata* | 5625 | 5532 | 6131 | 5790 | 6062 | 5857 | 9622 | 16241 | 16106 |
| **Japanese Spanish mackerel** | *Scomberomorus niphonius* | 19220 | 28080 | 34227 | 38161 | 28733 | 27172 | 31999 | 47005 | 58397 |
| **Red seabream** | *Pagrus major* | 5046 | 5293 | 2706 | 386 | 1082 | 943 | 2737 | 5528 | 3844 |
| **Grouper** | *Epinephelus* | 1777 | 1283 | 1222 | 1236 | 1229 | 2063 | 2030 | 2401 | 2212 |
| **Daggertooth pike conger** | *Muraenesox cinereus* | 27725 | 27479 | 36102 | 40617 | 44531 | 56087 | 77701 | 103829 | 111613 |
| [**Filefish**](http://www.fishbase.org/ComNames/CommonNameSummary.php?autoctr=304284) | *Thamnaconus* | 100662 | 196436 | 158128 | 92993 | 62015 | 29275 | 35072 | 39493 | 62111 |
| **Chub mackerel** | *Scomber japonicus* | 157449 | 100783 | 102956 | 111267 | 99474 | 121444 | 156791 | 131670 | 155938 |
| **Japanese scad** | *Decapterus maruadsi* | 124018 | 102802 | 135138 | 197525 | 212739 | 9220 | 171396 | 220974 | 253147 |
| **Japanese anchovy** | *Engraulis japonicus* | -- | 4576 | 22 | 15634 | 3835 | 237049 | 52168 | 27665 | 12752 |
| **South American pilchard** | *Sardinops sagax* | -- | 4153 | 2350 | 4326 | 15406 | 5110 | 1162 | 384 | 210 |
| **Japanese barracuda** | *Sphyraena japonica* | -- | 100 | 157 | 57 | 228 | 342 | 367 | 1151 | 1133 |
| **Pacific herring** | *Clupea pallasii* | -- | -- | -- | -- | -- | -- | -- | -- | -- |
| **Golden threadfin bream** | *Nemipterus virgatus* | -- | -- | -- | -- | -- | -- | -- | -- | -- |
| **Pacific cod** | *Gadus macrocephalus* | -- | -- | -- | -- | -- | -- | -- | -- | -- |
| **Silver croaker** | *Pennahia argentata* | -- | -- | -- | -- | -- | -- | -- | -- | -- |
| **Yellow drum** | *Nibea albiflora* | -- | -- | -- | -- | -- | -- | -- | -- | -- |
| **Mi-iuy croaker** | *Miichthys miiuy* | -- | -- | -- | -- | -- | -- | -- | -- | -- |
| **Baby croaker** | *Collichthys* | -- | -- | -- | -- | -- | -- | -- | -- | -- |
| **Horsehead tilefish** | *Branchiostegus japonicus* | -- | -- | -- | -- | -- | -- | -- | -- | -- |
| **Pacific sandlance** | *Ammodytes personatus* | -- | -- | -- | -- | -- | -- | -- | -- | -- |
| **Tuna** | *Thunnus* | -- | -- | -- | -- | -- | -- | -- | -- | -- |
| **Japanese jack mackerel** | *Trachurus japonicus* | -- | -- | -- | -- | -- | -- | -- | -- | -- |
| **Flathead grey mullet** | *Mugil cephalus* | -- | -- | -- | -- | -- | -- | -- | -- | -- |
| **Subtotal** | | 736309 | 800535 | 851281 | 922328 | 942802 | 968757 | 1166096 | 1461807 | 1455184 |
| **Mixed fish** | | 631237 | 680071 | 728024 | 741381 | 866784 | 1007737 | 1278806 | 1580138 | 1755856 |
| **Total catch** | | 1367546 | 1480606 | 1579305 | 1663709 | 1809586 | 1976494 | 2444903 | 3041945 | 3211040 |

**Continue**

| **Common name** | **Scientific name** | **Year** | | | | | | | | |
| --- | --- | --- | --- | --- | --- | --- | --- | --- | --- | --- |
|  |  | **1997** | **1998** | **1999** | **2000** | **2001** | **2002** | **2003** | **2004** | **2005** |
| **Large yellow croaker** | *Larimichthys crocea* | 7548 | 9952 | 9304 | 5893 | 4377 | 4387 | 13842 | 13481 | 9013 |
| **Yellow croaker** | *Larimichthys polyactis* | 54797 | 77913 | 96171 | 115844 | 82705 | 90002 | 78130 | 84987 | 78448 |
| **Largehead hairtail** | *Trichiurus lepturus* | 670232 | 718107 | 760080 | 823749 | 781941 | 733126 | 734825 | 773422 | 686740 |
| **Pomfret** | *Pampus* | 128351 | 154530 | 172968 | 187641 | 187707 | 201821 | 187915 | 189416 | 210371 |
| **Elongate ilisha** | *Ilisha elongata* | 6099 | 17312 | 14513 | 15980 | 20145 | 19237 | 26028 | 27561 | 26562 |
| **Japanese Spanish mackerel** | *Scomberomorus niphonius* | 76177 | 126234 | 143031 | 135968 | 137344 | 125249 | 78751 | 77678 | 86994 |
| **Red seabream** | *Pagrus major* | 13274 | 12028 | 10967 | 23716 | 15385 | 34560 | 32929 | 31082 | 35904 |
| **Grouper** | *Epinephelus* | 5255 | 6413 | 6735 | 7322 | 9191 | 9566 | 12875 | 10392 | 11891 |
| **Daggertooth pike conger** | *Muraenesox cinereus* | 115732 | 144500 | 136889 | 114463 | 122753 | 130499 | 142922 | 163323 | 141496 |
| [**Filefish**](http://www.fishbase.org/ComNames/CommonNameSummary.php?autoctr=304284) | *Thamnaconus* | 126647 | 65913 | 64624 | 55396 | 60101 | 35445 | 53671 | 58136 | 114953 |
| **Chub mackerel** | *Scomber japonicus* | 164847 | 127304 | 141178 | 120024 | 160020 | 183258 | 205649 | 234234 | 215324 |
| **Japanese scad** | *Decapterus maruadsi* | 174801 | 221425 | 229368 | 174680 | 219174 | 236371 | 305288 | 280210 | 294244 |
| **Japanese anchovy** | *Engraulis japonicus* | 134396 | 315701 | 170437 | 157557 | 162011 | 121241 | 186954 | 175157 | 155034 |
| **South American pilchard** | *Sardinops sagax* | 16786 | 822 | 1333 | 388 | 1152 | 1027 | 38024 | 40146 | 107143 |
| **Japanese barracuda** | *Sphyraena japonica* | 11910 | 11333 | 8013 | 14311 | 13271 | 12973 | 25648 | 33293 | 31178 |
| **Pacific herring** | *Clupea pallasii* | 5618 | 1130 | 0 | 226 | 445 | 173 | 7120 | 3721 | 8188 |
| **Golden threadfin bream** | *Nemipterus virgatus* | 1047 | -- | 476 | 2453 | 972 | 1125 | 140071 | 8069 | 5153 |
| **Pacific cod** | *Gadus macrocephalus* | -- | -- | -- | -- | -- | -- | 2077 | 673 | 689 |
| **Silver croaker** | *Pennahia argentata* | -- | -- | -- | -- | -- | -- | 56225 | 57808 | 56300 |
| **Yellow drum** | *Nibea albiflora* | -- | -- | -- | -- | -- | -- | 26628 | 28381 | 38595 |
| **Mi-iuy croaker** | *Miichthys miiuy* | -- | -- | -- | -- | -- | -- | 22930 | 19576 | 15922 |
| **Baby croaker** | *Collichthys* | -- | -- | -- | -- | -- | -- | 149886 | 162341 | 145764 |
| **Horsehead tilefish** | *Branchiostegus japonicus* | -- | -- | -- | -- | -- | -- | 22002 | 17774 | 20465 |
| **Pacific sandlance** | *Ammodytes personatus* | -- | -- | -- | -- | -- | -- | 61015 | 44923 | 49424 |
| **Tuna** | *Thunnus* | -- | -- | -- | -- | -- | -- | 23203 | 27694 | 30572 |
| **Japanese jack mackerel** | *Trachurus japonicus* | -- | -- | -- | -- | -- | -- | 32927 | 6094 | 10008 |
| **Flathead grey mullet** | *Mugil cephalus* | -- | -- | -- | -- | -- | -- | 10082 | 19766 | 28311 |
| **Subtotal** | | 1713514 | 2010615 | 1966085 | 1955609 | 1978692 | 1940058 | 2677615 | 2589338 | 2614686 |
| **Mixed fish** | | 1766038 | 1846331 | 1869294 | 1869256 | 1840120 | 1861803 | 1142205 | 1092940 | 1150618 |
| **Total catch** | | 3479552 | 3856946 | 3835380 | 3824866 | 3818812 | 3801862 | 3761243 | 3682278 | 3765304 |

**Continue**

**Continue**

| **Common name** | **Scientific name** | **Year** | | | | | | | | |
| --- | --- | --- | --- | --- | --- | --- | --- | --- | --- | --- |
|  |  | **2006** | **2007** | **2008** | **2009** | **2010** | **2011** | **2012** | **2013** | **2014** |
| **Large yellow croaker** | *Larimichthys crocea* | 4192 | 3823 | 5818 | 4003 | 4431 | 3960 | 4482 | 4816 | 5144 |
| **Yellow croaker** | *Larimichthys polyactis* | 83897 | 86004 | 93932 | 97901 | 105678 | 119891 | 112629 | 97855 | 104483 |
| **Largehead hairtail** | *Trichiurus lepturus* | 725082 | 540144 | 659065 | 664478 | 694746 | 637084 | 618435 | 607710 | 595443 |
| **Pomfret** | *Pampus* | 193411 | 175991 | 186115 | 181205 | 177314 | 175860 | 161973 | 143652 | 141259 |
| **Elongate ilisha** | *Ilisha elongata* | 27035 | 18535 | 28606 | 26577 | 25262 | 23808 | 23280 | 24570 | 23697 |
| **Japanese Spanish mackerel** | *Scomberomorus niphonius* | 80326 | 105279 | 112333 | 112596 | 124901 | 119613 | 119016 | 121759 | 123395 |
| **Red seabream** | *Pagrus major* | 46453 | 39903 | 43926 | 61395 | 63995 | 68960 | 65004 | 67834 | 68448 |
| **Grouper** | *Epinephelus* | 15603 | 16382 | 16979 | 16388 | 18645 | 19802 | 18358 | 18887 | 20857 |
| **Daggertooth pike conger** | *Muraenesox cinereus* | 238168 | 125940 | 141169 | 146913 | 148042 | 147814 | 153736 | 153122 | 156574 |
| [**Filefish**](http://www.fishbase.org/ComNames/CommonNameSummary.php?autoctr=304284) | *Thamnaconus* | 86839 | 69875 | 82555 | 96659 | 91808 | 93547 | 86451 | 88754 | 91164 |
| **Chub mackerel** | *Scomber japonicus* | 235017 | 173734 | 256626 | 232486 | 303767 | 358834 | 311159 | 306802 | 303096 |
| **Japanese scad** | *Decapterus maruadsi* | 315839 | 300085 | 362088 | 320875 | 335098 | 337425 | 352259 | 342097 | 357335 |
| **Japanese anchovy** | *Engraulis japonicus* | 152112 | 136594 | 150464 | 69424 | 120292 | 129177 | 142606 | 140518 | 72991 |
| **South American pilchard** | *Sardinops sagax* | 43055 | 38770 | 55904 | 32558 | 35973 | 44178 | 36627 | 34333 | 37825 |
| **Japanese barracuda** | *Sphyraena japonica* | 26957 | 20716 | 22834 | 23704 | 26557 | 24665 | 22851 | 21933 | 21550 |
| **Pacific herring** | *Clupea pallasii* | 20177 | 5934 | 12539 | 8533 | 8401 | 5614 | 5039 | 5230 | 4159 |
| **Golden threadfin bream** | *Nemipterus virgatus* | 6519 | 5349 | 13206 | 12562 | 11508 | 13278 | 13750 | 14440 | 15317 |
| **Pacific cod** | *Gadus macrocephalus* | 1095 | 1148 | -- | -- | -- | -- | -- | -- | -- |
| **Silver croaker** | *Pennahia argentata* | 54495 | 43859 | 63286 | 66500 | 67098 | 66275 | 63440 | 64162 | 57374 |
| **Yellow drum** | *Nibea albiflora* | 38711 | 34475 | 37943 | 48311 | 42748 | 41327 | 43057 | 41627 | 41607 |
| **Mi-iuy croaker** | *Miichthys miiuy* | 14217 | 22899 | 26590 | 26693 | 27841 | 25885 | 31602 | 61964 | 53887 |
| **Baby croaker** | *Collichthys* | 145414 | 117395 | 141458 | 145850 | 154899 | 191167 | 199807 | 191109 | 211093 |
| **Horsehead tilefish** | *Branchiostegus japonicus* | 16089 | 14113 | 20729 | 15168 | 15507 | 17714 | 20387 | 20623 | 19999 |
| **Pacific sandlance** | *Ammodytes personatus* | 47767 | 41664 | 40357 | 39893 | 43414 | 47978 | 49890 | 48338 | 51248 |
| **Tuna** | *Thunnus* | 43332 | 31862 | 7005 | 8632 | 9757 | 10610 | 7901 | 8175 | 8933 |
| **Japanese jack mackerel** | *Trachurus japonicus* | 9080 | 5239 | 9975 | 8926 | 8676 | 12014 | 10664 | 11523 | 11778 |
| **Flathead grey mullet** | *Mugil cephalus* | 17432 | 12317 | 20410 | 26580 | 27835 | 40251 | 33495 | 35255 | 45810 |
| **Subtotal** | | 2688314 | 2188029 | 2611912 | 2494810 | 2694193 | 2776731 | 2707898 | 2677088 | 2644466 |
| **Mixed fish** | | 1010749 | 909470 | 429736 | 772907 | 719506 | 789119 | 858286 | 879467 | 931910 |
| **Total catch** | | 3699063 | 3097499 | 3041648 | 3267717 | 3413699 | 3565850 | 3566184 | 3556555 | 3576376 |
