# Supplementary material for: Fisheries impacts on China's coastal ecosystems: Unmasking a pervasive ‘fishing down’ effect
Source: PLoS One. 2017 Mar 7;12(3):e0173296. doi: 10.1371/journal.pone.0173296 (PMC5340396; doi:10.1371/journal.pone.0173296)
Supplement: S2 Table — (DOCX) [file pone.0173296.s002.docx]

**S2 Table. Estimated growth and mortality parameters for 10 commercially exploited fishes in China’s seas in the 2000s (based on Liang and Pauly [1]).**

| **Common name** | **Scientific name** | **TL_∞_ (cm)** | **K estimation**  **(year^-1^)** | **M**  **(year^-1^)** | **F**  **(year^-1^)** | **L_c_ (cm)** |
| --- | --- | --- | --- | --- | --- | --- |
| **Largeheadhairtail** | *Trichiurus lepturus* | 152.4 | 0.38 | 0.54 | 1.45 | -- |
| **Yellow croaker** | *Larimichthys polyactis* | 28.8 | 0.49 | 0.86 | 1.45 | 4.90 |
| **Japanese grenadier anchovy** | *Coilia nasus* | 42.5 | 0.35 | 0.68 | 0.70 | 9.35 |
| **Japanese anchovy** | *Engraulis japonicus* | 18.4 | 0.51 | 1.00 | 0.93 | 4.60 |
| **Japanese scad** | *Decapterus maruadsi* | 32.7 | 0.89 | 1.45 | 0.44 | 7.19 |
| **Chub mackerel** | *Scomber japonicus* | 45.0 | 0.26 | 0.59 | 1.35 | 8.10 |
| **Pacific rudderfish** | *Psenopsis anomala* | 27.7 | 0.41 | 0.85 | 0.98 | 2.49 |
| **Osbeck's grenadier anchovy** | *Coilia mystus* | 26.0 | 0.54 | 1.12 | 0.88 | 7.28 |
| **Scaly hairfin anchovy** | *Setipinna taty* | 21.2 | 0.31 | 0.70 | 0.60 | 2.97 |
| **Bombay-duck** | *Harpadon nehereus* | 35.7 | 0.62 | 1.12 | 1.22 | 5.36 |
| **Mean** |  | -- | -- | -- | 1.00 | 5.8 (without *Trichiuruslepturus*) |

Note: TL_∞_ and K for the first five species in the table were estimated using the ELEFAN software [2, 3] and length frequency data; TL_∞_ for the last five species were also estimated using ELEFAN, because the length-frequency data of these five species, which covered only a narrow range of sizes, were not suitable for the estimation of K. Then K was estimated from ${\overline{\text{Ø}}}_{\text{m}}^{\text{,}}$= log(K)+2·log(L_∞_)_,_ where ${\overline{\text{Ø}}}_{\text{m}}^{\text{,}}$ is the mean of $\text{Ø}_{\text{m}}^{\text{'}}$-values estimated from different stocks of the same, or closely related species, based on L_∞_ and K pairs in FishBase ([www.fishbase.org](http://www.fishbase.org)); natural mortality (M) here was estimated from the empirical formula [4]: log(M) = -0.0066 – 0.279·log(L_∞_)+ 0.6543·log(K) + 0.4634·log(T); L_c_ and Z were estimated using ELEFAN; F was obtained from F= Z-M.

**References**

1. Liang C, Pauly D. Growth and mortality of exploited fishes in China’s coastal seas and their uses for yield-per-recruit analyses. Journal of Applied Ichthyology. 2017; Forthcoming.

2. Pauly D. A review of the ELEFAN system for analysis of length-frequency data in fish and aquatic invertebrates. In: Pauly D, Morgan GR, editors. Length-based methods in fisheries research. ICLARM Conference Procceedings 13;1987. pp. 7-34.

3. Pauly D. Beyond our original horizons: the tropicalization of Beverton and Holt. Reviews in Fish Biology and Fisheries. 1998; 8:307-334.

4. Pauly D. On the interrelationships between natural mortality, growth parameters, and mean environmental temperature in 175 fish stocks. Journal du Conseil. 1980;39(2):175-192.
