# Supplementary material for: Fisheries impacts on China's coastal ecosystems: Unmasking a pervasive ‘fishing down’ effect
Source: PLoS One. 2017 Mar 7;12(3):e0173296. doi: 10.1371/journal.pone.0173296 (PMC5340396; doi:10.1371/journal.pone.0173296)
Supplement: S3 Table — (DOCX) [file pone.0173296.s003.docx]

| **Common name** | **Scientific name** | **Trophic level** |
| --- | --- | --- |
| **Large yellow croaker** | *Larimichthys crocea* | 3.7 |
| **Yellow croaker** | *Larimichthys polyactis* | 3.6 |
| **Largehead hairtail** | *Trichiurus lepturus* | 4.4 |
| **Pomfret** | *Pampus* | 3.7 |
| **Elongate ilisha** | *Ilisha elongata* | 3.8 |
| **Japanese Spanish mackerel** | *Scomberomorus niphonius* | 4.5 |
| **Red seabream** | *Pagrus major* | 3.7 |
| **Grouper** | *Epinephelus* | 4.0 |
| **Daggertooth pike conger** | *Muraenesox cinereus* | 4.4 |
| [**Filefish**](http://www.fishbase.org/ComNames/CommonNameSummary.php?autoctr=304284) | *Thamnaconus* | 3.4 |
| **Chub mackerel** | *Scomber japonicus* | 3.4 |
| **Japanese scad** | *Decapterus maruadsi* | 3.4 |
| **Japanese anchovy** | *Engraulis japonicus* | 3.1 |
| **South American pilchard** | *Sardinops sagax* | 2.8 |
| **Japanese barracuda** | *Sphyraena japonica* | 4.2 |
| **Pacific herring** | *Clupea pallasii* | 3.2 |
| **Golden threadfin bream** | *Nemipterus virgatus* | 4.0 |
| **Pacific cod** | *Gadus macrocephalus* | 4.2 |
| **Silver croaker** | *Pennahia argentata* | 3.5 |
| **Yellow drum** | *Nibea albiflora* | 3.5 |
| **Mi-iuy croaker** | *Miichthys miiuy* | 3.5 |
| **Baby croaker** | *Collichthys* | 3.5 |
| **Horsehead tilefish** | *Branchiostegus japonicus* | 3.4 |
| **Pacific sandlance** | *Ammodytes personatus* | 3.1 |
| **Tuna** | *Thunnus* | 4.5 |
| **Japanese jack mackerel** | *Trachurus japonicus* | 3.4 |
| **Flathead grey mullet** | *Mugil cephalus* | 2.5 |

**S3 Table. Trophic levels for the studied 22 species and 5 genera**
